# Resiliency as a Protective Factor Against the Negative Impact of Adverse Childhood Experiences on Suicidality

**DOI:** 10.1007/s40653-026-00948-6

**Published:** 2026-07-30

**Authors:** Martie Thompson, Chloe Child, Casey Wilcox

**Affiliations:** Appalachian State University, Boone, United States

**Keywords:** Resiliency, Suicide attempts, Adverse childhood experiences, Add Health

## Abstract

Suicide is a leading cause of death in the United States. Suicide risk is higher among those who have experienced more adverse childhood events (ACEs). Yet the impact of ACEs on suicide risk could potentially be buffered by individual-level psychological characteristics. The purpose of this study was to test whether resiliency moderated the association between ACEs and suicide attempts. Using prospective data from a large, nationally representative sample of 8,671 respondents surveyed over 30 years, we found that although each additional ACE increased the odds for making a suicide attempt by 56%, these odds were significantly lower when resilience was high. Post hoc probing of the significant interaction term indicated that when ACEs were high, but resiliency was low, the probability that a participant had made a suicide attempt was 4.4%, compared to 1.6% when ACEs were high, but resiliency was also high. Our findings on the importance of resiliency in promoting mental health indicate the need for programs designed to promote resiliency across different settings.

## Suicide as a Public Health Problem

Suicide is a significant public health problem and has been a leading cause of death in the United States for the past 70years (2008 (Vital and Health Statistics; CDC, 2026). Rates of suicide increased 37% between 2000 and 2018, then decreased 5% between 2018 and 2020 before increasing again to their peak in 2022, with an age-adjusted rate per 100,000 of 14.2 (CDC, 2025). In 2023, there was a suicide death every 11 min in the United States, resulting in over 49,000 deaths (CDC, 2025). Suicide deaths represent the tip of the iceberg. Data from 2023 indicate that in addition to the 49,316 suicides, there were 1.5 million attempts, 3.7 million who made a suicide plan, and 12.8 million people who had serious thoughts about suicide (CDC, 2026). Thus, for every death by suicide, there are approximately 31 suicide attempts and 234 serious suicide ideations.

One of the most significant predictors of suicide is a prior attempt (Coon et al., 2024). Although most people (approximately 90%) who attempt suicide do not eventually die by suicide (Owens et al., 2002), over half of those who die by suicide have made a prior attempt (Ribeiro et al., 2016). These findings reveal the importance of understanding risk and protective factors for nonfatal suicidal attempts.

### Risk Factors of Suicide Behaviors

An extensive body of literature has identified many risk factors for suicidal behaviors, including mental health issues such as depression and anxiety, as well as stressful and traumatic life events (Franklin et al., 2017; Werbart et al., 2021). Adverse childhood experiences (ACEs) have also specifically been linked to an increased risk for suicidal behaviors (Choi et al., 2017; Dube et al., 2001; Ng et al., 2018; Thompson & Kingree, 2022). The various types of ACEs that have been shown to increase risk for suicidal behaviors include experiencing physical, sexual, and/or emotional abuse and neglect (Devries et al., 2014; Miller et al., 2013; Thompson & Kingree, 2022), having a parent with alcohol problems (Thompson et al., 2017; Thompson & Kingree, 2022), having a parental in jail during childhood (Dube et al., 2001; Thompson & Kingree, 2022), losing a parent to death (Guldin et al., 2015; Rostila et al., 2016; Thompson & Kingree, 2022), as well as having a family history of suicide (Brent et al., 2015; Feigelman et al., 2016; Thompson & Kingree, 2022). Further, research indicates that the accumulation of ACEs is associated with an increased risk for suicide attempts, such that those who experienced three or more ACEs were three times more likely to have attempted suicide than those who did not experience any ACEs (Thompson et al., 2019).

### Resiliency as a Protective Factor

Research has revealed several important risk factors for suicidality, including ACES, yet less research has focused on protective factors. One protective factor that this study seeks to investigate is resiliency. The term resilience refers to the ability to recover from or adjust easily to misfortune or change (Merriam- Webster, n.d.). This definition implies that resiliency can be conceptualized as a buffer against traumatic life experiences, thereby reducing their negative impact. A meta-analysis of 60 studies on the role of resiliency indicated that it was associated with better mental health outcomes, such as depression and anxiety (Hu et al., 2015). Although less attention has focused on the moderating or stress-buffering role of resilience in the presence of adversity, studies suggest that an individual’s level of resiliency can impact how they respond to adverse or traumatic events (Johnson et al., 2011). That is, a traumatic event may not have the same impact on everyone who experiences it. Rather, an individual’s degree of resiliency can buffer against some of the negative consequences of an adverse experience. In the Hu et al. (2015) meta-analysis, the effect sizes between resilience and mental health were significantly stronger for those experiencing adversity compared to those not experiencing adversities.

The purpose of our study was to determine if the prospective association between ACEs and suicide attempts was moderated by resiliency. We hypothesized that higher levels of ACEs would be prospectively associated with a higher likelihood of suicide attempts, and that suicide risk would be attenuated when resiliency was high. We used a nationally representative sample to generalize the results to the US population. We also took advantage of a longitudinal research design, such that we could examine the long-term protective impact of resiliency in the face of childhood trauma. Although the primary (i.e., universal) prevention of ACEs is ideal, this study can help inform secondary (i.e., selective) and tertiary (i.e., treatment) prevention efforts to reduce the negative sequelae of ACEs.

## Method

### Data Source and Sampling Procedures

We used restricted data from the National Longitudinal Study of Adolescent to Adult Health (Add Health, Harris, 2018; Harris et al., 2019). The Add Health study has followed a nationally representative sample of adolescents for approximately 30 years, from adolescence through emerging adulthood, to middle adulthood. The Add Health study was approved by the University of North Carolina School of Public Health Institutional Review Board using guidelines based on the Code of Federal Regulations on the Protection of Human Subjects 45CFR46. Local IRB approval for the secondary analysis of the restricted data was also obtained. Add Health participants provided written informed consent for participation in all aspects of the study. Participants were surveyed for the first time in 1994–1995, and then re-surveyed five additional times, 1, 7, 14, 22, and 30 years later, for a total of six waves of data collection. Details about study design, sampling, and survey procedures, and response rates are available on the Add Health website (http://www.cpc.unc.edu/addhealth).

The Add Health research design entailed multistage sampling and clustering of students withing schools. Thus, we needed to account for this in order to obtain unbiased variance estimates and to generalize our findings to the population. Thus, we restricted our sample to individuals with valid sampling weights to ensure our findings were nationally representative. The weight variable we used was recommended for longitudinal data with Wave 1 in-home survey participants who also completed surveys at Waves 4, 5, and 6 (Chen & Harris, 2020). This resulted in an analytic sample of 8,671 participants. The sample was evenly divided by sex at birth, with 50% males and 50% females. At Wave 5, participants were 33–43 years old, and at Wave 6, they were 39–51 years old.

### Measures

#### Dependent Variables

Respondents were asked at Waves 5 and 6 “During the past 12 months, how many times have you actually attempted suicide?” A dichotomous variable was created that reflected whether a respondent had attempted suicide at least once in the past year at either Wave 5 or Wave 6.

### Predictor Variables

We created a cumulative measure of ACEs by summing the number of eight different retrospectively reported adverse childhood experiences. In Add Health, these ACE items were reported at different times across the prospective study. However, we were able to ensure that incidents counted as an ACE occurred before the respondent was 18 years of age. Physical abuse, assessed at Wave 4, measured if respondents had been hit with a fist, kicked, or thrown on the floor, into a wall, or downstairs by a parent or adult caregiver more than once before they were 18 years of age (Brumley et al., 2017; Smith et al., 2018). Sexual abuse, assessed at Wave 4, measured if a parent or other adult caregiver had touched them in a sexual way, forced them to touch him or her in a sexual way, or forced them to have sexual relations before the age of 18. Emotional abuse, assessed at Wave 4, measured if a parent or other adult caregiver had said things that hurt their feelings or made them feel unwanted or unloved six or more times before their 18th birthday. We used a cut point of six or more times to increase the measure’s specificity in reflecting moderate-to‐severe emotional abuse (Scheidell et al., 2017; Smith et al., 2018). Neglect, assessed at Wave 3, measured if a parent or other adult caregiver had not taken care of their basic needs before sixth grade (Brumley et al., 2017). Parental death, assessed at Waves 1 and 2, measured if respondents had experienced the death of either parent during childhood. Parental incarceration, assessed at Wave 4, measured if a respondent’s biological mother or father had spent time in jail or prison before the youth was 18 years of age. Parental alcoholism, assessed at Wave 1 when the respondents were 12 to 17 years of age, measured if the child’s biological mother and/or biological father had alcoholism. Family history of suicide attempts, assessed at Waves 1 and 2, measured if a family member had tried to kill themselves during the past 12 months.

### Moderator: Resilience

We used the 13-item Adolescent to Adult Health Resilience Instrument (Montoya-Williams et al., 2020) to assess resiliency at Wave 4 when the respondents were on average 24–32 years old. The scale developers selected questions in Add Health that that were similar to items on other validated resilience scale (e.g., Connor Davidson Resilience Scale). The scale showed good psychometric properties and good construct validity, as indicated by its correlation with the Center for Epidemiologic Studies Depression Scale, which has been used to validate other resilience scales (Montoya-Williams et al., 2020). Items assess personal competence (e.g., “there is little I can do to change the important things in my life”), coping and isolation (e.g., “I often feel isolated from others;” “in the last 30 days, I have often felt unable to control the important things in my life”), and optimism (e.g., “overall, I expect more good things to happen to me than bad”). Items were transformed into z-scores prior to computing a mean score, since the response formats varied across items. Higher scores indicate greater resiliency (alpha = 0.79).

### Data Analytic Strategy

We computed weighted descriptive statistics to examine the prevalence of our study variables. We used SPSS (Version 29) Complex Samples to conduct logistic regressions that tested the moderating role of resiliency in the association between ACEs and suicide attempts. We first ran a model with measures of ACEs and resiliency, while controlling for sex and age. Then, we added an interaction term between resiliency and the respective ACE to the model to determine if resiliency buffered the association of ACEs with suicide attempts. Variance estimates were adjusted to account for the complex sample design. As noted previously, we applied sample weights so we could generalize our findings to the population. We used SPSS PROCESS (version 4.2) to conduct post hoc probing of significant interactions.

## Results

### Descriptive Statistics

Weighted frequencies indicated that 2.5% of the sample had attempted suicide at Waves 5 or 6. The number of ACEs reported by the sample ranged from 0 to 6. Based on weighted frequencies, a little over half of the sample (54.3%) reported no ACEs, and approximately one-quarter (25.4%) reported one ACE. Of the remaining respondents, 11.9% experienced two ACEs, 5.8% experienced three ACEs, 1.9% experienced four ACEs, 0.6% experienced five ACEs, and 0.1% reported six ACEs. The most common ACEs were experiencing emotional abuse (16.0%), having a parent with an alcohol problem (14.1%), experiencing physical abuse (12.9%), having a parent in jail (10.1%), experiencing neglect (7.2%), having someone in the family attempt or complete suicide (7.1%), experiencing sexual abuse (5.2%), and losing a parent to death as a child (4.9%).

### Logistic Regressions

Accounting for the complex sample design, weighting the data, and controlling for age and sex, results revealed that respondents higher in resiliency had a significantly lower odds of attempting suicide (AOR = 0.31; 95% CI = 0.24–0.41). This finding indicates that the odds of someone making a suicide attempt decreased by 69% for each unit increase in resiliency. The higher the number of ACEs, the higher the odds of attempting suicide in middle adulthood (AOR = 1.56; 95% CI = 1.33–1.83). This finding indicates that the odds of making a suicide attempt increased 56% for each additional ACE experienced.

**Fig. 1:**
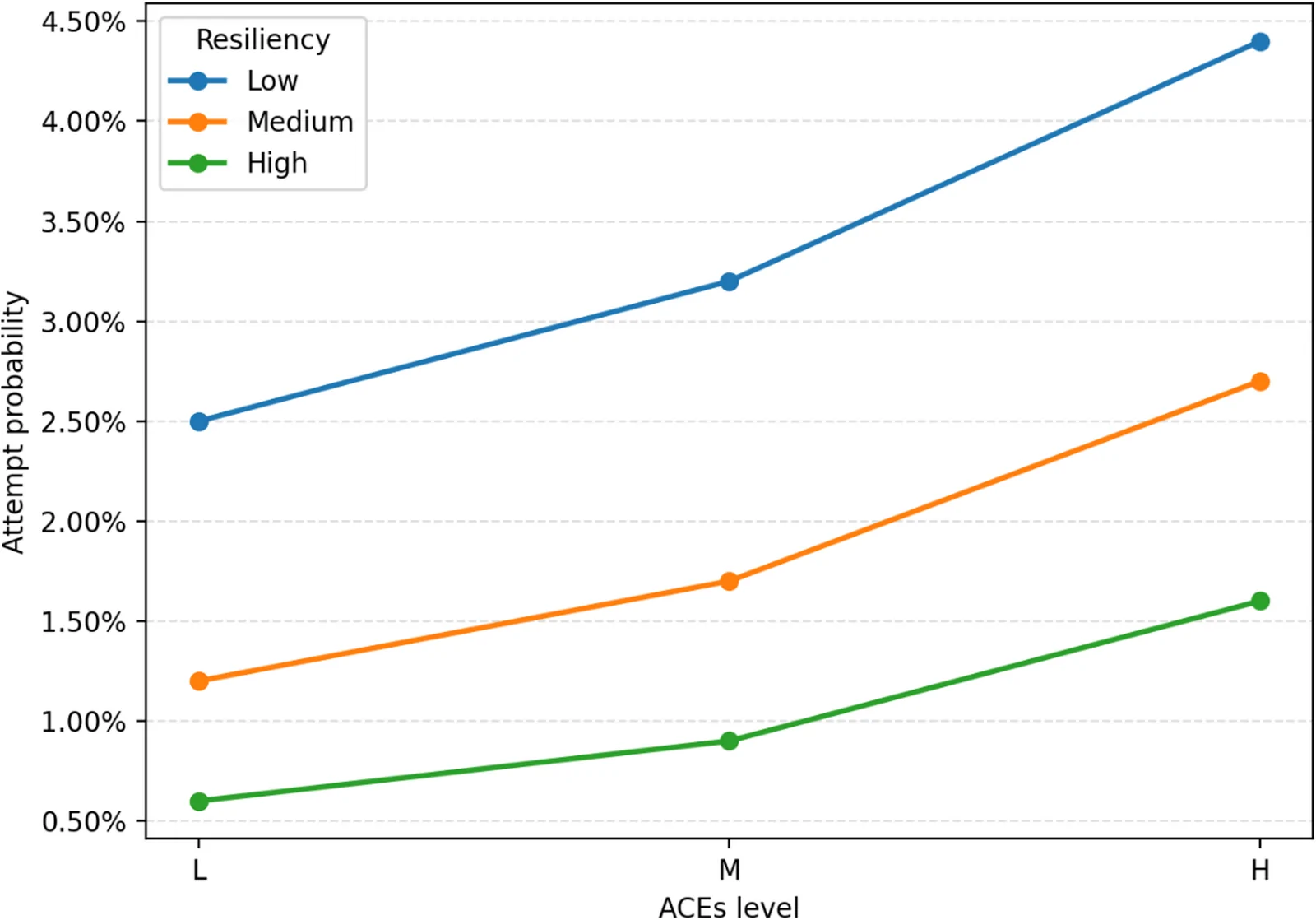
Plot of interaction between ACES and resiliency in predicting the probability of a suicide attempt

Of relevance to our study’s aims, resiliency significantly moderated the association between ACEs and the odds of a suicide attempt, indicating that the association between ACEs and suicide risk varied depending on a participant’s level of self-reported resiliency (parameter coefficient = 0.21; SE = 0.10, *p* =.04). As shown in Table 1 and in Fig. 1, post hoc probing of the significant interaction conducted using SPSS PROCESs (Hayes, 2018), indicated that when ACEs were high but resiliency was low, the probability that a participant had made a suicide attempt was 4.4%, compared to 1.6% when ACEs were high but resiliency was also high. When ACEs were low, but resilience was also low, the probability of a suicide attempt was 2.5%.

**Table 1:** Conditional effects of ACES on suicide risk at varying levels of the moderator

ACEs	Resiliency (moderator)	Probability of suicide attempt
Low	Low	.025
Medium	Low	.032
High	Low	.044
Low	Medium	.012
Medium	Medium	.017
High	Medium	.027
Low	High	.006
Medium	High	.009
High	High	.016

## Discussion

This study found that higher levels of ACEs were associated with a higher likelihood of suicide attempts up to 22–30 years later. The suicide risk was attenuated when resiliency was high. In fact, the probability of a suicide attempt when ACEs were high, yet resiliency was also high, was lower than when ACEs were low, but resilience was also low. The greatest odds of a suicide attempt were when ACEs were high, and resiliency was low.

These findings converge with previous research showing that an accumulation of ACEs is associated with a higher risk for suicidality (Thompson et al., 2019). Yet this study extends this research base by demonstrating that the risk conferred by ACEs extends for many years, as our data assessed for suicide attempts when respondents were in middle adulthood and the ACEs occurred prior to age 18. However, it is important to note that there are other factors that could also account for the association between ACEs and suicide risk.

Our finding that resilience was associated with a lower likelihood of suicide attempts also parallels prior research that has shown main effects of resiliency on mental health. Additionally, our findings converge with those of the Hu et al. (2015) meta-analysis, in that resiliency had a greater impact on mental health when experiencing adversity. In our sample, the difference in probability for suicide attempts was 47% greater when resiliency was high than when it was low.

Our study had several strengths worth noting. First, the sample was nationally representative and thus findings are generalizable to the US population. Second, the longitudinal research design enabled us to determine whether resiliency had a protective effect against childhood trauma 20–26 years later. Third, we used a comprehensive measure of ACEs that mirrored most of the ACEs assessed in the original Kaiser study (Felitti et al., 1998) and in the CDC ACE measure (Swedo et al., 2024).

Despite these strengths, our study had some limitations. The question about suicide attempts was only asked of those respondents who reported having seriously considered suicide. This could have led to misclassifying those who had attempted suicide impulsively (i.e., without serious prior consideration) as nonattempters. Also, suicide attempts were assessed with a past year reporting period. Consequently, our study may have misclassified people as nonattempters who may have attempted suicide at points in time other than the year prior to Wave 5 and the year prior to Wave 6. However, these measurement issues would have made it harder to find significant differences between attempters and their non-attempting counterparts. Additionally, we focused on suicide attempts rather than deaths. However, suicide attempts are important to study because a nonfatal suicide attempt is the strongest predictor of death by suicide (Bostwick et al., 2016), and risk factors for suicide attempts and completions are similar (Gould & Kramer, 2001). Lastly, because ACEs were assessed retrospectively, we may have underestimated the extent of ACEs, especially if a respondent was very young when experiencing the ACE.

Although the primary prevention of ACEs is ideal, this study suggests that secondary and tertiary prevention efforts to reduce the negative sequelae of ACEs are warranted. Strategies that promote resiliency skills and resilient characteristics (i.e., autonomy, self-efficacy, coping, optimism, sense of belonging) can help offset suicide risk in the presence of ACEs. Further, our results indicate that these strategies would be beneficial to anyone regardless of their experience with ACEs, as higher levels of resiliency were associated with a lower suicide attempt risk for the whole sample. Interventions to foster resiliency could take place in homes, school settings, and health care settings. Parents, teachers, and health professionals should be mindful of the importance of these resiliency skills and consider asking their children, students, and patients about their resiliency skills and offer suggestions for bolstering them.

In terms of interventions with parents, researchers have consistently found support for the role parenting behaviors can play in promoting resilience and well-being in children (Masten & Palmer, 2019). In a review of the effects of parenting prevention programs designed to promote resiliency in children and adolescents, Sandler et al. (2015) reported promising results. Specifically, programs such as the New Beginnings Program and the Family Bereavement Program not only promoted effective parenting and better parent-child relationships but also had positive impacts on mental health up to 15 years later.

There have also been positive results reported for school-based programs designed to promote student resiliency, self-efficacy, and coping skills. (Cai et al., 2025). In a review of the effectiveness of multiple types of school-based interventions (multicomponent, counseling, interpersonal regulation, mental health promotion, protective factors, leadership, support, and self-efficacy), 74% of programs reported a significant increase in resilience (Llistosella et al., 2023). In an Australian study that used a quasi-experimental design to test the effects of a universal, group-based, prevention program, significant increases in self-efficacy and coping skills were observed 2 months after the program (McAllister et al., 2018).

There is limited research on efforts to promote youth resiliency in health care settings (Vaughn & DeJonckheere, 2021), perhaps due to the primary focus in clinical practice to assess and treat problems rather than to assess strengths and link patients to resources. Researchers have suggested that clinicians assess youth’s relationships with caregivers, family environment, and access to potential resources in their schools, neighborhoods, and faith-based communities and provide information about accessing resiliency-promoting resources and supports (Jiang et al., 2022).

In summary, our study indicated that higher levels of ACEs were associated with a higher likelihood of suicide attempts up to 22–30 years later, but that suicide risk was attenuated when resiliency was high, suggesting the important role of resiliency in promoting mental health. Future research should investigate potential risk attenuators in addition to resiliency and focus on the development and evaluation of interventions designed to promote resiliency across different types of settings.

## Data Availability

We used the restricted Add Health data for this study. The restricted-use data are available by contractual agreement to qualified researchers who demonstrate a commitment to maintaining strict data security and limited access. Visit Add Health webpage for more information: https://addhealth.cpc.unc.edu/data/restricted-use-data/restricted-use-contracts/.
